# Improving patient outcomes with regenerative medicine: How the Regenerative Medicine Manufacturing Society plans to move the needle forward in cell manufacturing, standards, 3D bioprinting, artificial intelligence‐enabled automation, education, and training

**DOI:** 10.1002/sctm.19-0389

**Published:** 2020-03-28

**Authors:** Joshua Hunsberger, Carl Simon, Claudia Zylberberg, Preveen Ramamoorthy, Thomas Tubon, Ram Bedi, Kurt Gielen, Caitlin Hansen, Lynn Fischer, Jed Johnson, Priya Baraniak, Behzad Mahdavi, Taciana Pereira, Michael Hadjisavas, Shannon Eaker, Cameron Miller

**Affiliations:** ^1^ Regenerative Medicine Manufacturing Society Winston‐Salem North Carolina USA; ^2^ National Institute of Standards and Technology Gaithersburg Maryland USA; ^3^ Akron Biotech Boca Raton Florida USA; ^4^ HealthTell University of Colorado School of Medicine Aurora Colorado USA; ^5^ Madison College Madison Wisconsin USA; ^6^ University of Washington Seattle Washington USA; ^7^ Medace Maastricht Netherlands; ^8^ RoosterBio Ballenger Creek Maryland USA; ^9^ Title21 Health Solutions Pleasanton California USA; ^10^ Nanofiber Solutions Hilliard Ohio USA; ^11^ OrganaBio, LLC South Miami Florida USA; ^12^ Lonza Group Ltd Basel Switzerland; ^13^ Allevi Philadelphia Pennsylvania USA; ^14^ QIAGEN Hilden Germany; ^15^ GE Healthcare Marlborough Massachusetts USA; ^16^ Design Interactive Orlando Florida USA

**Keywords:** stem cells, technology, tissue engineering, tissue regeneration

## Abstract

The Regenerative Medicine Manufacturing Society (RMMS) is the first and only professional society dedicated toward advancing manufacturing solutions for the field of regenerative medicine. RMMS's vision is to provide greater patient access to regenerative medicine therapies through innovative manufacturing solutions. Our mission is to identify unmet needs and gaps in regenerative medicine manufacturing and catalyze the generation of new ideas and solutions by working with private and public stakeholders. We aim to accomplish our mission through outreach and education programs and securing grants for public‐private collaborations in regenerative medicine manufacturing. This perspective will cover four impact areas that the society's leadership team has identified as critical: (a) cell manufacturing and scale‐up/out, respectively, for allogeneic and autologous cell therapies, (b) standards for regenerative medicine, (c) 3D bioprinting, and (d) artificial intelligence‐enabled automation. In addition to covering these areas and ways in which the society intends to advance the field in a collaborative nature, we will also discuss education and training. Education and training is an area that is critical for communicating the current challenges, developing solutions to accelerate the commercialization of the latest technological advances, and growing the workforce in the rapidly expanding sector of regenerative medicine.

## Graphical abstract

Depicted are key areas of focus for the Regenerative Medicine Manufacturing Society. Advancing each of these areas will assist in moving the needle forward to improve patient outcomes with regenerative medicine.

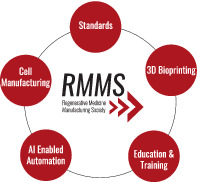

**Significance statement**

The Regenerative Medicine Manufacturing Society highlights focus areas to advance the field in a collaborative nature through working groups. These focus areas include cell manufacturing, standards for regenerative medicine, 3D bioprinting, and artificial intelligence‐enabled automation. These working groups will help disseminate this knowledge through future publications, as well as by identifying and developing education and training programs. Education and training programs can disseminate knowledge on current challenges and new technology innovations, as well as train the next generation workforce that will assist in making therapies the next standard of care.

## 1 INTRODUCTION

Understanding the clinical landscape and manufacturing challenges in cell‐based therapies^1^ is a critical first step to bringing these advanced therapies to the patient. We can then apply this knowledge to more complex tissue‐ and organ‐based therapies to manufacture these envisioned regenerative medicine products at commercial scale. Manufacturing is the most critical driver to truly translate regenerative medicine products into the marketplace thereby ensuring patient access. Developing manufacturing solutions, however, is no easy task. The Regenerative Medicine Manufacturing Society (RMMS) is poised to assist with moving the needle and developing these manufacturing solutions for the field through shared knowledge and collaboration. RMMS is the first and only professional society dedicated specifically toward advancing manufacturing solutions for the field of regenerative medicine. RMMS is assembling stakeholders from industry, academia, government, and nonprofit organizations to build working groups to advance areas critical to manufacturing with specific emphasis on scale up of cell manufacturing, standards, 3D bioprinting, and artificial intelligence (AI)‐enabled automation. Standards could be developed including documentary standards, reference materials, or reference data. These working groups will help evaluate the current landscape of work already done in both regenerative medicine and related fields such as cell therapy manufacturing, identify gaps, and propose solution spaces for the field to focus on. The leadership team has emphasized that RMMS will not reinvent the wheel, rather this society will bring together key leaders to work collaboratively. These perspective reports developed by RMMS working groups and the leadership team will be published annually in *Stem Cell Translational Medicine*, the official journal for RMMS. The society will also work to develop education and training programs to build competencies in all aspects of regenerative medicine (from tissue/cell harvest, transport, cell/tissue manufacturing, packaging, quality, regulatory, distribution, infusion/implant). These competencies can build the next workforce in regenerative medicine that industry and academic translational programs desperately need to improve patient outcomes in hitherto unimaginable ways.

## 2 CELL MANUFACTURING AND SCALE‐UP/OUT

Scale‐up cell manufacturing is an important workflow for regenerative medicine manufacturing. A recent perspective article examines the resources, initiatives, and regulatory pathways to advance regenerative medicine manufacturing.^2^ A critical challenge facing the field is developing processes and platform technologies that can cost effectively scale‐up/out the expansion of cells for allogenic (on the order of billions) or autologous (patient specific).^3^, ^4^ Upstream and downstream manufacturing processes are also critical to scale‐related analysis, as each is dependent on maintaining the desired phenotype throughout the manufacturing process.^5^ RMMS is using its working groups to work collaboratively to identify available technologies and highlight potential gaps, in addition, to identifying and proposing new platform technologies that can address some of the gaps in scale‐up/out challenges. In the area of critical raw materials used in the manufacturing process, groups are working on advancing media development for clinical cell manufacturing designed for scalability, efficiency, and productivity that should greatly reduce process development time as well as reduce adventitious agent testing.^6^

In the area of compliant manufacturing facilities, groups are developing cleanroom alternatives for regenerative medicine facilities that can provide standalone current good manufacturing practices (cGMP) environments at lower costs, be placed in existing laboratories or renovated space, be decontaminated through highly automated processes, be modular as to accommodate a multistep manufacturing process and eliminate the potential for cross contamination, be compliant with regulatory agencies; with the ultimate goal of aseptically closing the manufacturing process to drive‐down cost of goods (COGs). Electrochemical sensors are in development for in‐line continuous monitoring of glucose and lactate concentration in cell culture^7^ as well as the development of automated cell counting and cell sorting instruments. There is also considerable work being done on developing 3D bioreactors for more efficient and productive expansion and maturation of cells and bioengineered tissues and organs for allogenic and fully automated single‐use patient‐scale bioreactors for autologous applications. Considerations for current and future advances in bioreactors for cell therapies has been reviewed.^8^, ^9^ Moreover, considering COGs for producing a cell therapy product has also been reviewed.^10^

The manufacturing cost is a key consideration in advancing regenerative medicine therapies to the clinic and widespread commercialization. To this end, cleanroom alternatives for regenerative medicine facilities that focus on the minimization/elimination of a number of pain points including, but not limited to cGMP environments at dramatically lower costs, cGMP manufacturing in existing, “unqualified” spaces, decontamination through automated processes, and modular designs so as to accommodate multistep manufacturing processes would greatly benefit the industry as a whole. An emerging paradigm in cGMP manufacturing that addresses a number of current bottlenecks is the concept of flexible, turn‐key cleanrooms. Such multitenant GMP facilities would allow companies to manufacture their own products, retaining their intellectual property, with access to quality and manufacturing systems and services needed to do so. By availing themselves of this resource, companies can avoid the capital expense of building a facility or relinquishing control of their manufacturing process to a third party while gaining ready access to raw materials and expertise in GMP manufacturing, quality control, and regulatory affairs. This will advance the path to clinical translation and commercialization by driving down costs and reducing manufacturing failure rates associated with third party technology transfer.

RMMS working groups can assist further by integrating current advances in this cell manufacturing and scale‐up/out space and work with industry partners to develop commercial solutions for technology gaps when available or by integrating different technologies to improve efficiency. These new or integrated solutions could also arise from new technology development supported from federal grants that could be targeted to address these critical gaps. The development of cell‐free regenerative medicine solutions such as exosome therapy, which have clear advantages in terms of manufacturing, transport, and overall cost of production, will also be closely monitored and reported by this group.^11^

## 3 STANDARDS

There are numerous scientific considerations for regulating a cell therapy^12^ and 3D printed medical product^13^ where the development of documentary standards, reference materials, or reference data will accelerate process development for regenerative medicine products. Development of industry standards is important for the advancement of regenerative medicine. Many organizations are working in this exciting area including the National Institute of Standards and Technology, the Standards Coordinating Body for Gene, Cell, and Regenerative Medicines and Cell‐Based Drug Discovery (SCB), ASTM International, the International Organization for Standardization (ISO), Foundation for the Accreditation of Cellular Therapy, the National Academies of Sciences Engineering Medicine, International Society for Cell and Gene therapy (ISCT), International Society for Stem Cell Research, and the IEEE Standards Association. The RMMS working group in standards hopes to collaborate with many of these above organizations in the development of safety and quality standards specifically for regenerative medicine manufacturing.

There are many opportunities to create standard guides and test methods in the field, from cell counting in the manufacturing process to identification testing of raw materials, to the creation of validated or even compendial assays to assess the critical quality attributes (CQAs) of cellular raw materials. Cellular starting materials manufactured with validated processes, using qualified raw materials, and following cGMP and regulatory requirements can in turn become starting materials used in cell therapy products.^14^

RMMS working groups will develop vetted protocols that can be used to validate CQA and critical process parameters (CPPs) of a regenerative medicine product along a manufacturing process continuum based on the desired cell types. Vetted protocols can be developed collaboratively with RMMS members and then shared in perspective articles for critical review. These vetted protocols could also be developed into commercial assays that could be readily used by the entire field to validate different regenerative medicine manufacturing workflows. These vetted protocols could also be used to optimize manufacturing processes in real time or terminate manufacturing runs early if desired CQAs were not met. Eventually, the vetted protocols could be used to develop documentary standard test methods at ASTM or ISO.

Furthermore, development of platforms that can screen these assay data and make them available remotely to mobile devices for real time monitoring are also being developed^15^ and could further improve their utility. Electronic quality systems can be leveraged to support compliance to standards, improve speed to market and advance U.S. Food & Drug Administration (FDA) approvals of regenerative medicine products. Clearly, standardization is a challenging area that will highly benefit from collaborative efforts and critical reviews from RMMS members.

## 4 3D BIOPRINTING

3D bioprinting is a platform technology for advancing regenerative medicine manufacturing. 3D bioprinting is envisioned to fabricate multifunctional drug‐delivery systems,^16^ scaffolds,^17^ prosthetics (eg, dental and orthopedic),^18^ organoids,^19^ tissues, and organs.^20^ The use of this technology has grown tremendously in the past 5 years, especially as the cost of devices dropped dramatically, paving the way for more widespread use. In addition, key industry players have developed software platforms that also help in democratizing the use of this technology due to easier‐to‐use interfaces.

However, developing out of the box customized bioinks that can be sold in tandem with this technology is lagging behind. Several groups are working on developing materials that can be used across different bioprinting platforms and tuned for specific applications.^21^ These materials, however, have demonstrated a trade‐off between printability and biological relevance. Some groups focus on plant‐based, easier‐to‐print bioinks, while others focus on ECM (extracellular matrix) component bioinks, that are more biologically relevant but also more difficult to print. Also, a supply chain of high‐quality bioinks with preservation of CQAs at later passage is necessary for scale‐up. In addition, ASTM is working on bioink standards, including printability of bioinks and biomaterials that could be used in automated biofabrication technology,^22^ and guidance on development of bioinks, including considerations such as material properties that assist with survival of cells within bioinks.^23^ Development of these types of standards can assist with defining key requirements needed to develop new bioinks.

Another limiting factor is the regulatory hurdle of developing combination products. Using a 3D bioprinter to create implants consisting of a scaffold and living cells will need to go through review by both device and biologic regulatory bodies. This results in long and expensive regulatory approval pathways compared with traditional implants. Early clinical opportunities may include external regenerative medicine targets such as cell‐based wound dressings. The RMMS working group will assist with understanding some of the regulatory challenges and technology gaps in this rapidly advancing area and highlight member‐developed solutions. We envision assisting with the advancement of commercial products with industry partners in this space based on recommendations from our working groups.

## 5 AI‐ENABLED AUTOMATION

Deployment of AI to automate regenerative medicine processes will likely be the most disruptive advance for regenerative medicine. This fact can be supported by major investments in this area from countries around the world including the United States,^24^ Canada,^25^ the United Kingdom,^26^ India,^27^ Germany,^28^ and China.^29^ While all these efforts do not directly integrate these advances in automation with regenerative medicine they could be easily adapted for such applications as the manufacturing workflows for a tissue engineered regenerative medicine technology for both autologous and allogeneic therapies have been described.^30^

The RMMS working group can assist with identifying critical manufacturing processes in this workflow that can be automated with current technology. Advances in automation have the potential to increase manufacturing efficiency and lower COGs while maintaining key manufacturing requirements, like closed systems, to minimize safety risks.^31^ Key areas for scale‐up are filtration, continuous centrifugation, automated vialing, and final product freezing. Comparability studies and standardization across these platforms are key to successful implementation of scale‐up and automated technology based on lot‐size and phase‐specific needs. AI may be especially helpful for analyzing images of the product during manufacturing to predict tissue function.^32^ This is indeed a very exciting and pivotal era. One might even say we are in a digital revolution in manufacturing for regenerative medicine. This revolution of digital manufacturing includes monitoring, analytics, and new computing capabilities combined with advances in AI‐enabled to revolutionize manufacturing workflows from product development to factory operations to supply chains to shipping and logistics.^33^ Digital manufacturing is no longer a figment of our imagination. It is present now and becoming part of biomanufacturing.

This combined term, digital biomanufacturing, is the next evolution where this process seeks to improve the manufacturing of biological entities (eg, cells, tissues, organs) that are known to be variable. Digital biomanufacturing can provide significant advances in regenerative medicine manufacturing by ensuring high quality and reproducibility of complex bioengineered tissues and organs that can be customized for patients to replicate digital magnetic resonance imaging (MRI) medical scan images using 3D computer models, machine learning and AI. Machine leaning is also gaining acceptance in biopharmaceutical manufacturing as a tool for bioprocess development and optimization^34^ where RMMS working groups may play a role in adapting some of these machine learning algorithms to provide real time data on biomanufacturing processes based on CQAs and CPPs defined for specific regenerative medicine products.

Applying AI to such data‐rich processes inherent in biomanufacturing a regenerative medicine‐based product can lead to sophisticated and predictive algorithms that are envisioned for numerous applications. Highlighting some of these applications include being able to optimize bioreactor design for cell and tissue expansion or use real time data feedback loops to optimize a process in an automated fashion to ensure a clinical product meets CQAs.^35^ RMMS can work in tandem to bring academia and industry together to automate manufacturing processes and develop commercial products that can make these solutions widely available to advance the field. Moreover, the working group can make recommendations on incorporating AI into this manufacturing workflow and connecting new startup companies and academic teams in different disciplines with applications for regenerative medicine manufacturing.

## 6 EDUCATION AND TRAINING

Education, training, and workforce readiness in the area of regenerative medicine are critical to expand the current talent pool with the requisite knowledge, skills, and abilities to safely and efficiently manufacture and deliver the regenerative medicine products and therapies of the future. Importantly, promoting career pathways in regenerative medicine also provides a vehicle for creating learning and dissemination networks to address the current challenges, latest advances, and solutions to rapidly adapt to the changing needs of this emergent sector. RMMS will collaborate with the National Science Foundation ATE Innova ATEBIO Center for Biotechnology to develop these learning networks across all education levels from K‐12, technical community colleges, 4‐year and postgraduate degrees (BS, MS, and PhD), and incumbent work professional development programs. We also envision developing hands‐on postgraduate level training workshops and internship programs.

RMMS would like to highlight education and training programs that our members currently have and subsequently identify gaps where we could assist in developing new training programs or expanding current ones to close these skill gaps. Areas to consider are hands‐on cell culture techniques, the latest manufacturing technology, analytical methods, and regulatory requirements. As standards are created, they can be fed into these types of programs to teach industry‐accepted methods early. We also envision working with innovators to build augmented reality training modules^36^ that can be applied toward certifications, retraining, collaboration, troubleshooting, and many other uses. Augmented reality will be another disruptive technology that can connect this field in new ways currently not being used. RMMS will actively seek to expand the representation of underserved, underrepresented, and diverse talent pool to drive the next generation of highly‐skilled technical workers.

## 7 CONCLUSIONS

In summary, RMMS is seeking to bring together the field of regenerative medicine through working groups to identify the current gaps and solution spaces. We will work collaboratively with partners to identify and grow new education and training programs. We are also seeking new global collaborations^37^ where we envision being able to attract additional talents that will assist our mission in advancing regenerative medicine through developing manufacturing solutions and advancements. Interested parties are encouraged to visit our RMMS website to learn more about the society and the working groups. We also had our open member's meeting at the World Stem Cell Summit and Phacilitate Leader's Conference on January 21, 2020, in Miami, FL. This meeting was a tremendous success in communicating future visions for RMMS, progress made by each of our working groups, and engaging new members to become strategically involved in this society to advance the field of regenerative medicine manufacturing.

## CONFLICT OF INTEREST

C.Z. declared employment and intellectual property rights with Akron Biotech and advisory role with CCRM, ARM Foundation, BioFlorida, ISCT, SCB. R.B. declared advisory role with PHC Corporation of North America, Konica Minolta, Inc. K.G. declared leadership position with Brightlands Chemelot Campus B.V. L.F. declared leadership position and stock ownership with Title21 Health Solutions. P.B. declared leadership position with RoosterBio Inc and equity interest in OrganaBio LLC. T.P. declared leadership position with Allevi, Inc. The other authors declared no potential conflicts of interest.

## AUTHOR CONTRIBUTIONS

J.H.: concept and design, manuscript writing, and final approval; C.S., C.Z., P.R., T.T., R.B., K.G., C.H., L.F., J.J., P.B., B.M., T.P., M.H., S.E., C.M.: manuscript writing.

## Data Availability

Data sharing is not applicable to this article as no new data were created or analyzed in this study.
